# Rare Circulating Cells in Familial Waldenström Macroglobulinemia Displaying the *MYD88* L265P Mutation Are Enriched by Epstein-Barr Virus Immortalization

**DOI:** 10.1371/journal.pone.0136505

**Published:** 2015-09-09

**Authors:** Maroulio Pertesi, Perrine Galia, Nicolas Nazaret, Maxime Vallée, Laurent Garderet, Xavier Leleu, Hervé Avet-Loiseau, Matthieu Foll, Graham Byrnes, Joel Lachuer, James D. McKay, Charles Dumontet

**Affiliations:** 1 Genetic Cancer Susceptibility, International Agency for Research on Cancer, Lyon, France; 2 Hospices Civils de Lyon, Pierre Bénite, France; INSERM 1052, CNRS 5286, CRCL, Lyon, France; University of Lyon, Lyon, France; 3 ProfilExpert, Lyon, France; 4 INSERM, UMR_S 938, Proliferation and differentiation of stem cells, Paris, France; AP-HP, Hôpital Saint Antoine, Département d'hématologie et de thérapie cellulaire, Paris, France; Sorbonne Universités, UPMC Univ Paris 06, Paris, France; 5 Service des maladies du sang, Hopital Huriez CHRU, Lille, France; Centre de Recherche INSERM U837, Facteurs de persistance des cellules leucémiques, Institut pour la Recherche sur le Cancer (IRCL), Lille, France; 6 Unité génomique du myélome, Institut Universitaire du Cancer de Toulouse—Oncopole, CRCT INSERM U1037, Toulouse, France; 7 Biostatistics, International Agency for Research on Cancer, Lyon, France; University of Nebraska - Lincoln, UNITED STATES

## Abstract

The *MYD88* L265P is a recurrent somatic mutation in neoplastic cells from patients with Waldenström Macroglobulinemia (WM). We identified the *MYD88* L265P mutation in three individuals from unrelated families, but its presence did not explain the disease segregation within these WM pedigrees. We observed the mutation in these three individuals at high allele fractions in DNA extracted from EBV-immortalized Lymphoblastoid cell lines established from peripheral blood (LCL), but at much lower allele fractions in DNA extracted directly from peripheral blood, suggesting that this mutation is present in a clonal cell subpopulation rather than of germ-line origin. Furthermore, we observed that the *MYD88* L265P mutation is enriched in WM families, detected in 40.5% of patients with familial WM or MGUS (10/22 WM, 5/15 MGUS), compared to 3.5% of patients with familial MM or MGUS (0/72 MM, 4/41 MGUS) (p = 10^−7^). The mutant allele frequency increased with passages *in vitro* after immortalization with Epstein-Barr virus (EBV) consistent with the *MYD88* L265P described gain-of-function proposed for this mutation. The *MYD88* L265P mutation appears to be frequently present in circulating cells in patients with WM, and MGUS, and these cells are amenable to immortalization by EBV.

## Introduction

Waldenström Macroglobulinemia (WM) is a rare B-cell lymphoproliferative disorder demonstrating an IgM monoclonal gammopathy. Monoclonal Gammopathy of Undetermined Significance (MGUS) is a premalignant plasma cell disorder that precedes the development of WM, Multiple Myeloma (MM) and other related Plasma cells disorders, with a risk of progression in the order of 1.5% per year.[1] Among lymphoid malignancies, WM displays the strongest familial predisposition to develop the disease or clonal abnormalities. In a series of 257 unrelated WM patients, Treon *et al*. observed a clustering of B-cell disorders among first-degree relatives of patients with WM, where nearly one in five WM patients had at least one first-degree relative with WM (5.1%), some form of hematological malignancy (11.7%) or Monoclonal Gammopathy of Unknown Significance (MGUS; 1.9%).[2] Similarly, in a large study of 2,144 WM/lymphoplasmacytic lymphoma patients and 8,279 population-based matched controls, Kristinsson *et al*. found that first-degree relatives presented a 20-fold risk of developing the same disease.[3] In addition, a recent large population-based study showed that the presence of family history is associated with significantly lower survival.[4] The existence of a familial predisposition is thus well documented in this disease.

*MYD88* has been found to be involved in the pathogenesis of sporadic Waldenström Macroglobulinemia (WM). Treon *et al*. found a recurrent somatic mutation in *MYD88*, L265P, in the bone marrow of 26 out of 30 Waldenström patients by whole genome sequencing, with an additional case identified by Sanger sequencing.[5] All patients with a family history were found to carry the mutation, while it was absent in the germ-line material. The *MYD88* L265P mutation has been shown to be a gain of function driver mutation in models such as ABC non-Hodgkin lymphoma cell lines, while activated MYD88 induces NF-kappa B signaling.[6,7]

Our study was initially designed to determine whether germ-line mutations in *MYD88* were present in familial cases of WM, and subsequently explored the presence of the missense *MYD88* L265P mutation at rare allele fractions within WM, MM families.

## Materials and Methods

### Patient samples

Families with at least two cases of Waldenström disease and/or IgM Gammopathy were enrolled for this study from across France. Targeted resequencing of the coding sequence of the *MYD88* gene using exon capture was performed on a total of 41 cases with IgM monoclonal component (27 WM, 14 IgM-MGUS) and 5 additional family members with non-IgM monoclonal component or full-blown multiple myeloma (4 IgG-MGUS, 1 IgA-myeloma) (S1 Fig). The ages at accrual for the WM cases are shown in S1 Table. In addition, deep Ion-Torrent based targeted resequencing for the *MYD88* L265P mutation was performed on a series of 74 multiple myeloma cases and 43 MGUS cases recruited from French families with reoccurrence of multiple myeloma/MGUS, and a series of 55 French sporadic lung cancer samples recruited as described elsewhere.[8] The study was approved by the Hospices Civils de Lyon institutional review board and participants signed an informed consent form. Peripheral blood was drawn and lymphoblastoid cell lines were established as previously described.[9] DNA from lymphoblastoid cell lines after several *in vitro* passages was extracted using QIAmp DNA Mini Kit (Qiagen) according to the manufacturer’s recommendations, and subsequently quantified with the Qubit fluorometer using dsDNA HS Assay (Life Technologies).

### Targeted resequencing

Targeted exon capture was performed using the HaloPlex Target Enrichment kit 1–500 kb (Agilent) according to the HaloPlex Target Enrichment System-Fast Protocol Version B. Briefly, the protocol consists of the following four steps: 1) Digestion of genomic DNA in eight different restriction reactions. 2) Hybridization of restricted fragments to probes whose ends are complementary to the target fragments. During hybridization, fragments are circularized and sequencing motifs, including index sequences, are incorporated. 3) Capture of target DNA using streptavidin beads and ligation of circularized fragments. 4) PCR amplification of captured target libraries.

250 ng of DNA was split among 8 different restriction digests each containing 2 restriction enzymes. The eight different digests corresponding to the same DNA sample were further pooled and used in the capture reaction with probes. HaloPlex probes are designed to hybridize selectively to fragments originating from target regions of the genome and to direct circularization of the targeted DNA fragments. During the hybridization process, Illumina sequencing motifs including index sequences (for sample multiplexing) are incorporated into the targeted fragments. Prior to pooling 48 samples, DNA was quantified using a Qubit Fluorometer (Life Technologies). Circularized fragments were then captured, ligated and amplified by PCR. Pooled libraries were further paired-end sequenced (150 bp reads) in a flow cell version 2 on a MiSeq instrument (Illumina, USA). Image analysis and base calling was performed using the Illumina RTA software version 1.13.48. The mean coverage was 375X.

### Deep Ion Torrent-based targeted sequencing

PCR primers were designed (F: 5’-ggttgaagactgggcttgtc-3’, R: 5’-gcgagtccagaaccaagatt-3’) using Primer 3 Plus to amplify an amplicon of 198bp spanning the L265P on genomic DNA. 30ng of DNA were amplified by PCR using AccuStartTaq DNA Polymerase (Quanta BioSciences) according to manufacturer’s instructions. Library preparation for Ion Torrent was performed using the NEBNext Fast DNA Library Preparation Kit (New England Biolabs), template preparation was done on the Ion OneTouch2 instrument using the Ion PGM Template OT2 200 Kit, followed by sequencing on an Ion Torrent PGM sequencer using the Ion PGM Sequencing 200 Kit v2 (Life Technologies).

### Bioinformatics analyses, Variant analyses

Raw reads were aligned onto the reference human genome (build hg19 from UCSC database) using Bowtie 2 (version 2.0.2). Bowtie was run in local mode: consequently it does not require that the entire read aligns on the reference sequence. Reads with poor qualities were filtered out: “Illumina Passing Filter” metric was used to perform quality filtering.[10] Sequencing output files were mapped on hg19 reference genome with BWA, and then sorted with Picard SortSam (http://picard.sourceforge.net). Picard Mark Duplicates was used to flag duplicate reads. Files were then locally realigned and base score recalibrated using the Genome Analysis Tool Kit (GATK).[11–13] Recalibrated files were used to call the variants. Both single nucleotide variants (SNV) and indels were called using GATK Unified Genotyper. All the called variants were then annotated with AnnoVar (version 2013 Aug 23).[14] Calls were subsequently filtered following a two-step approach. The first step consisted in pruning from the raw list of calls, variants that failed several quality control criteria (criteria as exonic status, segmental duplications, strand bias, different starting point of reads, quality of the call, coverage from NHLBI GO Exome Sequencing Project (ESP6500) (Exome Variant Server, NHLBI GO Exome Sequencing Project (ESP), Seattle, WA), frequency in ESP6500, frequency in 1000 genomes project, frequency in a panel of 500 normal TCGA.[15,16] The second step focused on functionality to keep only missense substitutions assessed as deleterious by either SIFT or PolyPhen.[17,18]

### Selection of samples for deep Ion Torrent sequencing, statistical analyses

55 Lung cancer samples were selected as reference samples in order to calculate the distribution of the mutant allele fractions observed by chance. In order to calculate the minimum allele fraction above which samples are considered “positive” for the *MYD88* L265P mutation, we first transformed the allelic fractions to log-odds which provided approximately normally distributed values. We then set the threshold as 6 standard deviations above the mean, after removing the 2 most extreme values at each end of the distribution. Exceeding this threshold corresponds to rejection of the presence of the *MYD88* L265P mutation at p<2x10-9.

We then applied this threshold to the Ion Torrent-based deep sequencing data on WM, MM and MGUS individuals. We did not consider individuals with a mean read depth lower than 500X, thus excluding 1 WM, 2 MM, and 2 MGUS individuals from MM families, that underperformed or failed due to technical problems. We then selected for analysis on DNA prior to EBV-immortalization (0p), after 4 passages (4p) and after 9 passages (9p) all 19 samples that were classed as “positive” after fulfilling the above mentioned criteria. For a subset of 13 individuals positive for the *MYD88* mutation, independent amplification and sequencing was performed for the 9p DNA sample to test the reproducibility of the allele fraction observation, and the results showed strong concordance (ICC = 0.84).

The proportion of variant alleles was regressed, within each sample, against the number of passages, using random effects negative binomial model with total alleles included as the exposure.

Tests of association of MYD88 mutation presence and case status (WM vs MM) used Fisher’s exact test.

## Results

We sequenced the *MYD88* gene (OMIM: 602170) using HaloPlex targeted capture on an Illumina MiSeq, in 41 cases with IgM monoclonal component (27 WM, 14 IgM MGUS), 5 additional family members with IgG monoclonal component or full-blown multiple myeloma (4 IgG-MGUS, 1 IgA-myeloma), and 2 healthy individuals, from 20 unrelated families with at least two cases of WM or IgM MGUS (S1 Table and S1 Fig). The *MYD88* L265P mutation (NM_002468.4) was first identified in LCLs from three individuals, 1 WM and 2 IgM MGUS cases, with allelic fractions of 43.4%, 55.7% and 22.6%, respectively. The presence of this mutation was further confirmed using an independent PCR amplification and subsequent targeted Sanger sequencing. These individuals originated from three unrelated families, and the mutation did not segregate with the disease within the families (S2 Fig). We subsequently undertook targeted Ion Torrent-based deep sequencing (>16,000X) for the *MYD88* L265P mutation in these 3 individuals using DNA extracted from blood samples not subject to EBV-immortalization (0p). The *MYD88* L265P was present in the non-immortalized DNA samples, but at considerably lower allelic fractions compared to DNA from LCLs (0.37%, 8.51% and 1.29%), suggesting that the *MYD88* L265P mutation was more likely to be of somatic, rather than germ-line, origin.

We subsequently determined the prevalence of the *MYD88* L265P mutation among WM, MM and MGUS patients from WM and MM families. We performed deep Ion Torrent-based sequencing (mean read depth 11,781X) for the *MYD88* L265P mutation using DNA extracted from LCLs after 9 passages *in vitro* (9p) in 38 out of 46 individuals from WM families described above (23 WM and 15 MGUS), and in 117 individuals from a collection of familial multiple myeloma families (74 MM and 43 MGUS). We defined individuals as “positive” for the *MYD88* L265P mutation when the proportion of the variant allele was in excess of the background error rate (0.47%), defined using 55 lung cancer samples (Materials and Methods, S2 Table). After removing 5 under-performing samples (1 WM, 2 MM and 2 MGUS from MM families) with a mean read depth lower than 500X, we identified 15 individuals (40.5%) from the WM families (10/22 WM, 5/15 MGUS) and 4 individuals (3.5%) from the MM families (4/41 MGUS including 2 IgM-MGUS, 0/72 MM) positive for the *MYD88* L265P variant. Individuals classified as carrying the *MYD88* mutation were more frequent in WM families in comparison to MM families (Fischer’s exact test P = 1.167× 10^−7^). Notably, 1 out of 55 individuals from the lung cancer series used as reference samples, was observed to have a *MYD88* L265P allelic fraction of 2.5%. It is worth to note that those lung cancer patients were not selected for the absence of WM, MM or MGUS, and posterior review of the clinical status confirmed the presence of MGUS in this patient.

**Fig 1:**
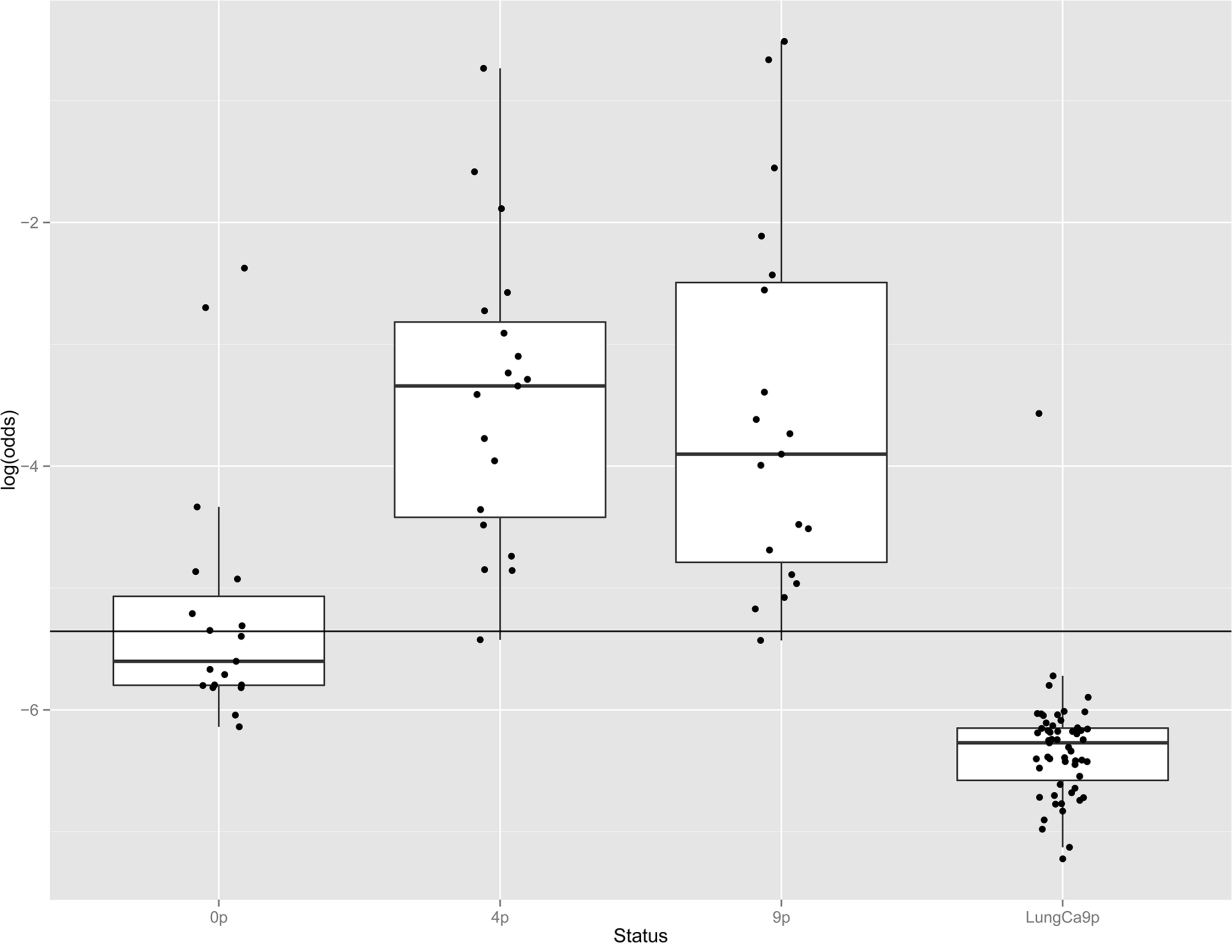
Distribution of *MYD88* L265P allele fractions in samples from patients with familial Waldenström Macroglobulinemia prior to or after immortalization by EBV, and in lymphoblastoid lines derived from patients with lung cancer.

We additionally considered all 19 individuals (10 WM and 5 IgM MGUS from WM families, 4 MGUS from MM families) identified above with significant MYD88 L265P mutation allele fractions and performed deep Ion Torrent-based sequencing (mean read depth 18,000X) in DNA extracted across three time points: from blood prior to EBV-immortalization (0p), from LCLs after 4 passages *in vitro* (4p) and 9 passages *in vitro* (9p), with the 9p points repeated for a subset of 13 samples. Eight out of 19 patients (42%) had detectable MYD88 L265P allele fractions above the detection threshold (AF 0.47–8.51%) prior to EBV-immortalization (0p), whereas 18 out of 19 had significant allele fractions at 4 and 9 passages *in vitro* (Table 1). The proportion of the MYD88 L265P allele fraction increased with *in vitro* passages (Fig 1) by a factor of 1.11 per passage (95% CI 1.07–1.16, p<0.001). The enrichment was more evident between the 0p and 4p steps (S3 Fig), by a factor of 1.46 per passage (95%Ci 1.33–1.56 p<0.001), whereas samples reached a plateau or even lowered between 4p and 9p steps (0.97, 95%Ci 0.92–1.08 per passage, p = 0.29) (Table 1).

**Table 1:** *MYD88* L265P allele fraction (AF %), given as the ratio of mutant/reference sequence reads, in samples prior to (0p) or after immortalization with EBV (4 and 9 passages *in vitro*).

Disease	Family	Individual	Type of component	prior to immortalization (0p)			4 *in vitro* passages (4p)			9 *in vitro* passages (9p)		
				AF (%)	Total	Alt	AF (%)	Total	Alt	AF (%)	Total	Alt
**Familial WM**	1	1	IgM MGUS	**0.77**	18037	138	**1.88**	22478	422	**1.81**	11923	216
	2	3	WM	**0.33**	23027	76	**3.79**	18091	685	**7.21**	25153	1814
	2	4	WM	**0.72**	22352	161	**3.60**	13306	479	**2.33**	13195	308
	3	6	IgG MGUS	**1.29**	19943	258	**17.02**	20819	3544	**17.47**	11066	1933
	18	12	IgG MGUS	**0.49**	13014	64	**0.87**	15566	135	**0.62**	18559	115
	11	14	WM	**0.30**	18473	56	**0.44**	16634	73	**0.44**	11912	52
	13	16	WM	**0.47**	18797	89	**2.25**	13533	304	**2.62**	19223	503
	14	19	IgM MGUS	**6.30**	17704	1116	**6.15**	17832	1097	**3.25**	15712	511
	14	20	WM	**0.34**	20620	71	**1.12**	14142	158	**0.75**	6563	49
	15	21	WM	**0.45**	14622	66	**7.08**	26112	1848	**8.09**	16425	1328
	11	34	WM	**0.37**	18208	67	**5.17**	11832	612	**33.96**	17552	5961
	16	35	WM	**0.22**	26473	57	**0.77**	16841	130	**1.12**	29768	334
	5	37	WM	**0.30**	28694	87	**3.42**	13437	459	**0.56**	23214	131
	5	45	IgM MGUS	**8.51**	16449	1400	**32.40**	22636	7335	**37.42**	20127	7531
	9	46	IgM MGUS	**0.30**	16520	49	**0.78**	32414	252	**0.91**	29725	271
**Familial MM**		A	IgM MGUS	**0.30**	34466	104	**4.32**	17563	758	**1.98**	24381	483
		B	IgM MGUS	**0.54**	22647	123	**3.19**	19072	609	**1.08**	48700	528
		C	IgG MGUS	**0.30**	12142	36	**13.17**	9677	1274	**10.79**	9449	1020
		D	IgA MGUS	**0.24**	15617	37	**1.27**	16424	208	**0.69**	9656	67

**AF: allele fraction**; Total: total number of reads; Alt: number of reads with the mutant allele; WM: Waldenström disease; MM: multiple myeloma

Analysis was performed on samples obtained from 19 patients (10 WM and 5 MGUS from WM families, 4 MGUS from MM families) found to carry the mutation, across three time points; from blood prior to EBV-immortalization (0p), from LCLs after 4 passages *in vitro* (4p) and 9 passages *in vitro* (9p), as well as in LCLs derived from 55 patients with lung cancer after 9 passages *in vitro* (LungCa9p).

The black bars in the box structures represent the median value and the black dots outside the upper and lower whiskers correspond to outliers. The horizontal black line corresponds to the 0.47% threshold, as defined by the distribution of allelic fractions observed in the lung cancer comparison group (see Materials and Methods). The outliers in the 0p sample group correspond to samples with high allelic frequency, while the outlier within the Lung Ca samples was found to be a patient with MGUS.

0p: prior to EBV-immortalization, 4p: after 4 passages *in vitro*, 9p: after 9 passages *in vitro*, LungCa9p: lung cancer reference samples after 9 passages *in vitro*.

## Discussion

Our study initially aimed to investigate whether germ-line genetic variants in the *MYD88* gene contribute to the genetic susceptibility to WM. We identified the presence of a rare *MYD88* L265P mutation in individuals from three unrelated families. However, the presence of this variant did not explain the familial aggregation as it did not segregate with the disease within the pedigrees. Furthermore, given the observed higher variant allele fractions following EBV-immortalization and cell culture in comparison to the uncultured blood, the *MYD88* L265P mutation does not appear to be involved in genetic susceptibility to WM and is more likely of somatic origin. As such, it appears that our observation of the *MYD88* L265P in DNA extracted from Lymphoblastoid cell lines is due to the presence of the mutation either in a sub-clone or in circulating free cell(s) (CFC), rather than a germline variant or cell free DNA. Indeed, the presence of clonotypic cells in peripheral blood is well described in WM. Smith *et al*. originally used flow cytometry to describe the presence of clonal cells in peripheral blood and suggested that it is a common finding in WM, while very rare in multiple myeloma (MM).[19] Kriangkum *et al*. observed preferential use of VH3/JH4 gene families in WM and expansion of polyclonal cells in peripheral blood.[20] Much more recently, the presence of somatic mutations in peripheral blood has been documented, suggesting that age-related clonal hematopoiesis is a common condition associated with an increased risk of hematologic cancer.[21] Our demonstration of circulating cells harboring the somatic *MYD88* L265P mutation in WM patients is consistent with these observations. However, primary cells rapidly apoptose in culture and EBV has generally been reported to infect but not to immortalize leukemic B lymphocytes.[22] It is thus unexpected that a *MYD88* positive cell population derived from a patient sample would be susceptible to immortalization by EBV and proliferate *in vitro* over prolonged periods of time, raising the possibility that a precursor bearing the mutation but susceptible to immortalization is present in the peripheral blood of patients and is being enriched with cell culture. Further description of the cellular characteristics of the *MYD88* L265P positive clones may be informative.

EBV-transformed lymphoblastoid cell lines have been shown to evolve during long-term culture. Lee *et al*. have reported that “late” (161 passages) cells compared to “early” (4 passages) displayed phenotypic changes involving the NF-kappa B pathway and carcinogenesis-related genes.[23] Additionally, the ability of EBV to immortalize B cells may be linked to P53 content.[24] A study in diffuse large B-cell lymphoma, showed that the MYD88 L265P mutant promoted cell survival by triggering IRAK-mediated NF-κB signaling, acting as a gain of function driver mutation.[6] Thus, the increasing allele fraction we observe subsequent to *in vitro* culture, and by inference the proportion of cells carrying the *MYD88* L265P variant, may be due to a survival advantage conveyed to cells bearing this mutation. Our observation suggests that the establishment of lymphoblastoid cell lines could be used to enrich for certain types of rare B cell subpopulations present in patient samples.

The *MYD88* L265P mutation is highly frequently observed in the bone marrow (BM) from patients with WM, and relatively frequently in the BM from MGUS patients, while absent in multiple myeloma patients.[25] In our series, we found this mutation to be significantly overrepresented in families with a family history of WM relative to families with a family history of MM, consistent with the observation in BM samples in previous studies. This was largely due to its absence in MM patients as opposed to its presence in MGUS. Additionally, we noted the presence of the *MYD88* L265P in patients with IgM or IgG-MGUS, as well as in MGUS patients from families with multiple myeloma.

Several studies have developed conventional and real-time allele-specific PCR assays (AS-PCR, ASO-RQ-PCR) for the identification of the *MYD88* L265P mutation in a rapid and sensitive manner, at a lower detection limit of 0.1% to 0.25% when using BM-isolated CD19+ selected cells or BM samples.[26, 27] Here, we used a combination of EBV-immortalized lymphoblastoid cell line culture and NGS techniques to detect this mutation in peripheral blood samples from WM and MGUS patients. Our findings suggest that cell culture when combined with sensitive mutation detection assays, may have potential as a relatively non-invasive method for detecting the *MYD88* L265P mutation.

One important limitation of our study is that LCLs derived from patients with lung cancer were used as reference samples in order to calculate the detection limit of the next generation sequencing based assay for the *MYD88* L265P mutation. While not available to the current study, LCLs from unaffected controls are necessary to establish a less biased estimate of the error rate of this assay, as well as the population prevalence of the *MYD88* L265P mutation under such conditions.

In conclusion, our findings do not support the presence of germ-line *MYD88* mutations in familial WM. However, our results show the presence of circulating cells susceptible to immortalization by EBV bearing the *MYD88* mutation in the peripheral blood of some patients with familial WM or IgM-MGUS. Additional studies are required to determine whether the presence of cells bearing the *MYD88* L265P is correlated with disease progression and outcome in patients with WM or MGUS.

## Supporting Information

S1 Fig Family trees from patients with familial Waldenström disease and/or IgM MGUS. (JPG)

S2 Fig Examples of pedigrees of families with peripheral blood cells harboring the *MYD88* L265P mutation using targeted resequencing on an Illumina MiSeq. DNA samples were extracted after 9 passages *in vitro* (9p), and the mutant allele fraction (%) is given as the ratio of mutant/reference reads. In the case of individual 45, DNA was extracted after 5 passages *in vitro* (5p).
(JPG)

S3 Fig Evolution of *MYD88* L265P allele fractions prior to and after immortalization by EBV. Analysis was performed on samples obtained from 19 patients positive for the *MYD88* L265P mutation, across three time points; from blood prior to EBV-immortalization (0p), and from LCLs after 4 passages *in vitro* (4p) and 9 passages *in vitro* (9p). The horizontal black line corresponds to the 0.47% threshold, as defined by the distribution of allelic fractions observed in the lung cancer comparison group (see Materials and Methods).
(JPG)

S1 Table Number and age of cases with monoclonal components within Waldenström Macroglobulinemia families. n.a.: not available, WM: Waldenström Macroglobulinemia, MGUS: Monoclonal Gammopathy of Unknown Significance. ^a^ Samples not available
(DOC)

S2 Table *MYD88* L265P allele fractions (AF %) in 55 lung cancer samples, given as the ratio of mutant/reference sequence reads. Total: total number of reads; Alt: number of reads with the mutant allele.
(DOCX)
