# Supplementary figures and images for: Rare Circulating Cells in Familial Waldenström Macroglobulinemia Displaying the MYD88 L265P Mutation Are Enriched by Epstein-Barr Virus Immortalization
Source: PLoS One. 2015 Sep 9;10(9):e0136505. doi: 10.1371/journal.pone.0136505 (PMC4564105; doi:10.1371/journal.pone.0136505)

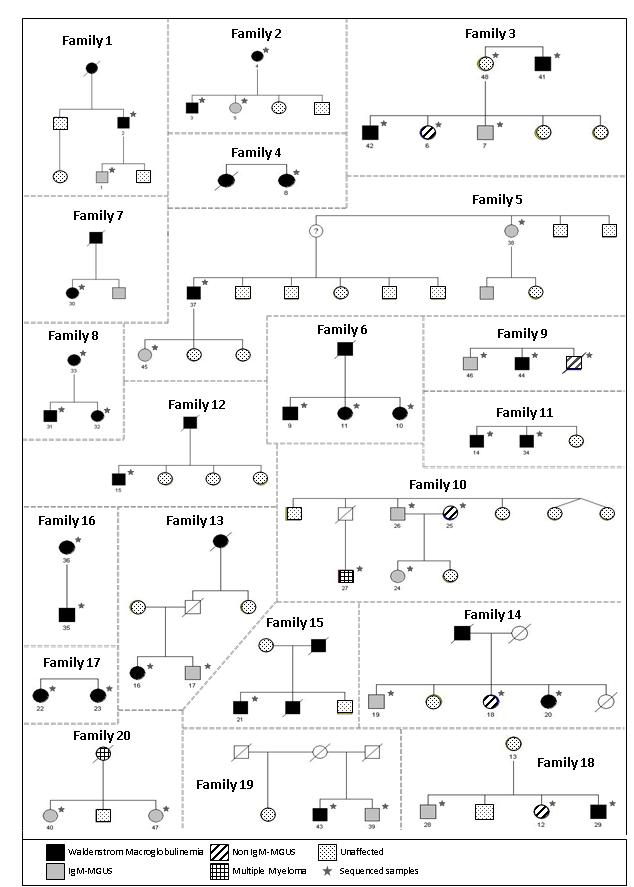

Supplement: S1 Fig — (JPG) [file pone.0136505.s001.jpg]

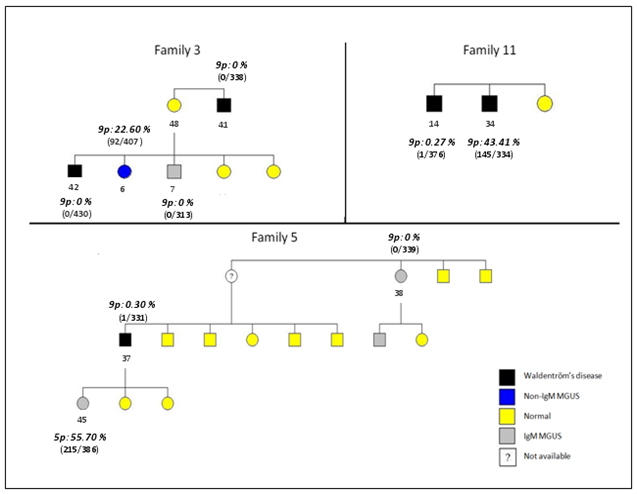

Supplement: S2 Fig — DNA samples were extracted after 9 passages in vitro (9p), and the mutant allele fraction (%) is given as the ratio of mutant/reference reads. In the case of individual 45, DNA was extracted after 5 passages in vitro (5p). (JPG) [file pone.0136505.s002.jpg]

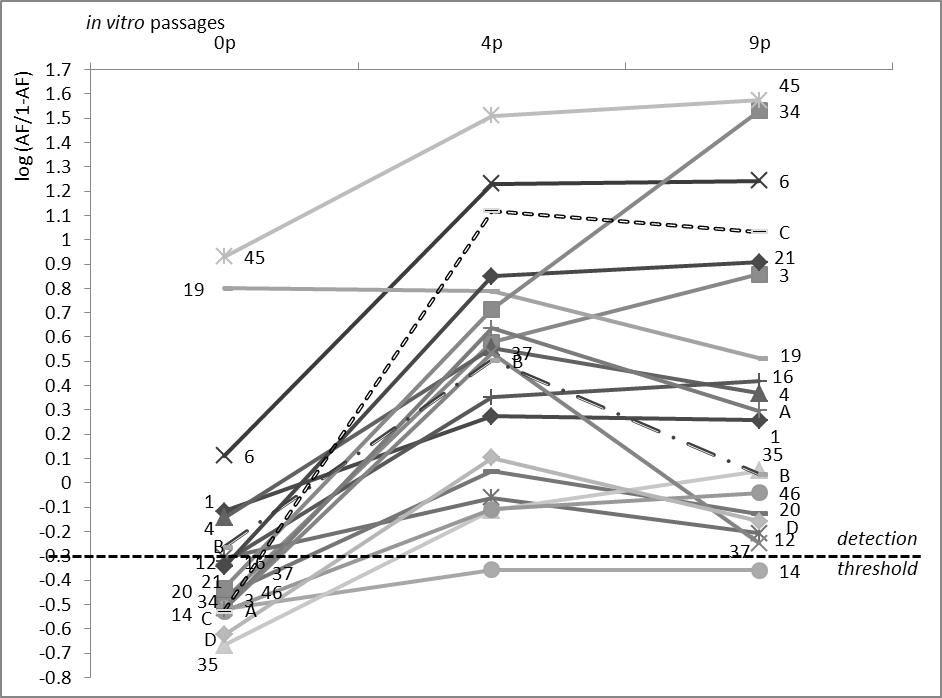

Supplement: S3 Fig — Analysis was performed on samples obtained from 19 patients positive for the MYD88 L265P mutation, across three time points; from blood prior to EBV-immortalization (0p), and from LCLs after 4 passages in vitro (4p) and 9 passages in vitro (9p). The horizontal black line corresponds to the 0.47% threshold, as defined by the distribution of allelic fractions observed in the lung cancer comparison group (see Materials and Methods). (JPG) [file pone.0136505.s003.jpg]
