# Supplementary material for: Rare Circulating Cells in Familial Waldenström Macroglobulinemia Displaying the MYD88 L265P Mutation Are Enriched by Epstein-Barr Virus Immortalization
Source: PLoS One. 2015 Sep 9;10(9):e0136505. doi: 10.1371/journal.pone.0136505 (PMC4564105; doi:10.1371/journal.pone.0136505)
Supplement: S1 Table — n.a.: not available, WM: Waldenström Macroglobulinemia, MGUS: Monoclonal Gammopathy of Unknown Significance. a Samples not available (DOC) [file pone.0136505.s004.doc]

| **Family** | **WM cases (Age)** | **IgM-MGUS cases (Age)** | **Non-IgM MGUS cases (Age)** | **Myeloma cases (Age)** | **All cases (Available)** |
| --- | --- | --- | --- | --- | --- |
| 1 | 2 (57/n.a.^a^) | 1 (33) | - | - | 3 (2) |
| 2 | 2 (84/62) | 1 (61) | - | - | 3 |
| 3 | 2 (76/61) | 1 (56) | 1 (49) | - | 4 |
| 4 | 2 (75, n.a.^a^) | - | - |  | 2 (1) |
| 5 | 1 (62) | 3 (79/54^a^/36) | - |  | 4 (3) |
| 6 | 4 (57/55/53/n.a.^a^) | - | - |  | 4 (3) |
| 7 | 2 (73/n.a.^a^) | 1 (71^a^) | - |  | 3 (1) |
| 8 | 3 (77/53/52) | - | - |  | 3 |
| 9 | 1 (62) | 1 (59) | 1 (n.a.^a^) |  | 3 (2) |
| 10 | - | 2 (82/54) | 1 (76) | 1 (55) | 4 |
| 11 | 2 (83/79) | - | - |  | 2 |
| 12 | 2 (56/n.a.^a^) | - | - |  | 2 (1) |
| 13 | 2 (57/n.a.^a^) | 1 (53) | - |  | 3 (2) |
| 14 | 2 (61/n.a.^a^) | 1 (74) | 1 (70) |  | 4 (3) |
| 15 | 3 (58/n.a.^a^) | - | - |  | 3 (1) |
| 16 | 2 (92/59) | - | - |  | 2 |
| 17 | 2 (85/83) | - | - |  | 2 |
| 18 | 1 (60) | 1 (71) | 1 (68) |  | 3 |
| 19 | 1 (66) | 1 (63) | - |  | 2 |
| 20 | - | 2 (65/64) | 1 (n.a.^a^) |  | 3 (2) |
| ***Total (available)*** | ***36 (27)*** | ***16 (14)*** | ***6 (4)*** | ***1 (1)*** | ***59 (46)*** |
