# Supplementary material for: Rare Circulating Cells in Familial Waldenström Macroglobulinemia Displaying the MYD88 L265P Mutation Are Enriched by Epstein-Barr Virus Immortalization
Source: PLoS One. 2015 Sep 9;10(9):e0136505. doi: 10.1371/journal.pone.0136505 (PMC4564105; doi:10.1371/journal.pone.0136505)
Supplement: S2 Table — Total: total number of reads; Alt: number of reads with the mutant allele. (DOCX) [file pone.0136505.s005.docx]

| **Sample Type** | **Sample name** | **AF (%)** | **Total number of reads** | **Alt reads** |
| --- | --- | --- | --- | --- |
| Lung Ca | C1 | 0.19 | 16596 | 31 |
| Lung Ca | C2 | 0.19 | 19270 | 37 |
| Lung Ca | C3 | 0.22 | 9823 | 21 |
| Lung Ca | C4 | 0.33 | 13065 | 43 |
| Lung Ca | C5 | 0.21 | 17398 | 36 |
| Lung Ca | C6 | 0.21 | 22784 | 49 |
| Lung Ca | C7 | 0.24 | 11366 | 27 |
| Lung Ca | C8 | 0.21 | 18944 | 39 |
| Lung Ca | C9 | 0.24 | 21396 | 51 |
| Lung Ca | C10 | 0.19 | 17549 | 34 |
| Lung Ca | C11 | 0.16 | 9152 | 15 |
| Lung Ca | C12 | 0.19 | 9267 | 18 |
| Lung Ca | C13 | 0.21 | 10609 | 22 |
| Lung Ca | C14 | 0.12 | 14684 | 18 |
| Lung Ca | C15 | 0.09 | 8601 | 8 |
| Lung Ca | C16 | 0.21 | 11024 | 23 |
| Lung Ca | C17 | 0.11 | 10465 | 12 |
| Lung Ca | C18 | 0.20 | 11808 | 24 |
| Lung Ca | C19 | 0.16 | 13585 | 22 |
| Lung Ca | C20 | 0.16 | 9888 | 16 |
| Lung Ca | C21 | 0.12 | 12732 | 15 |
| Lung Ca | C22 | 0.16 | 18346 | 29 |
| Lung Ca | C23 | 0.21 | 10880 | 23 |
| Lung Ca | C24 | 0.18 | 10771 | 19 |
| Lung Ca | C25 | 0.30 | 9601 | 29 |
| Lung Ca | C26 | 0.12 | 15728 | 19 |
| Lung Ca | C27 | 0.24 | 11051 | 27 |
| Lung Ca | C28 | 0.11 | 8342 | 9 |
| Lung Ca | C29 | 0.16 | 12886 | 21 |
| Lung Ca | C30 | 0.17 | 9519 | 16 |
| Lung Ca | C31 | 0.14 | 10449 | 15 |
| Lung Ca | C32 | 0.20 | 6833 | 14 |
| Lung Ca | C33 | 0.07 | 13709 | 10 |
| Lung Ca | C34 | 0.08 | 11223 | 9 |
| Lung Ca | C35 | 0.13 | 8197 | 11 |
| Lung Ca | C36 | 0.21 | 11097 | 23 |
| Lung Ca | C37 | 0.11 | 16635 | 19 |
| Lung Ca | C38 | 0.15 | 16941 | 26 |
| Lung Ca | C39 | 0.19 | 7796 | 15 |
| Lung Ca | C40 | 0.23 | 10141 | 23 |
| Lung Ca | C41 | 0.17 | 9052 | 15 |
| Lung Ca | C42 | 0.12 | 14938 | 18 |
| Lung Ca | C43 | 0.10 | 21925 | 22 |
| Lung Ca | C44 | 0.17 | 14357 | 24 |
| Lung Ca | C45 | 0.18 | 12081 | 22 |
| Lung Ca | C46 | 0.24 | 13556 | 32 |
| Lung Ca | C47 | 0.27 | 16782 | 46 |
| Lung Ca | C48 | 0.24 | 9864 | 24 |
| Lung Ca | C49 | 0.21 | 8943 | 19 |
| Lung Ca | C50 | 0.17 | 12686 | 21 |
| Lung Ca | C51 | 0.24 | 10450 | 25 |
| Lung Ca | C52 | 0.13 | 8771 | 11 |
| Lung Ca | C53 | 0.22 | 9451 | 21 |
| Lung Ca | C54 | 2.74 | 7687 | 211 |
| Lung Ca | C55 | 0.13 | 14595 | 19 |
